# The Investigation of Reducing PAHs Emission from Coal Pyrolysis by Gaseous Catalytic Cracking

**DOI:** 10.1155/2014/528413

**Published:** 2014-05-14

**Authors:** Yulong Wang, Ruifang Zhao, Chun Zhang, Guanlong Li, Jing Zhang, Fan Li

**Affiliations:** State Key Laboratory Breeding Base of Coal Science and Technology Co-founded by Shanxi Province and the Ministry of Science and Technology, Taiyuan University of Technology, Taiyuan 030024, China

## Abstract

The catalytic cracking method of PAHs for the pyrolysis gaseous products is proposed to control their pollution to the environment. In this study, the Py-GC-MS is used to investigate in situ the catalytic effect of CaO and Fe_2_O_3_ on the 16 PAHs from Pingshuo coal pyrolysis under different catalytic temperatures and catalyst particle sizes. The results demonstrate that Fe_2_O_3_ is effective than that of CaO for catalytic cracking of 16 PAHs and that their catalytic temperature corresponding to the maximum PAHs cracking rates is different. The PAHs cracking rate is up to 60.59% for Fe_2_O_3_ at 600°C and is 52.88% at 700°C for CaO. The catalytic temperature and particle size of the catalysts have a significant effect on PAHs cracking rate and CaO will lose the capability of decreasing 16 PAHs when the temperature is higher than 900°C. The possible cracking process of 16 PAHs is deduced by elaborately analyzing the cracking effect of the two catalysts on 16 different species of PAHs.

## 1. Introduction

Polycyclic aromatic hydrocarbons (PAHs) are hazardous organic pollutants widely existing in the environment. Many of them have the carcinogenic, teratogenic, and mutagenic effects which mainly come from incomplete combustion of fossil fuels and hydrocarbons or from the pyrolysis in their reducing process [1]. Although the amount of PAHs in environment is trace, it has become a great menace to human health because it distributes widely in the air, soil, water, and vegetation through continuous generation, transformation, migration, and degradation and will enter the human body through the respiratory tract, skin, and digestive tract. Consequently, the sources of PAHs have received considerable attention from people.

The coal pyrolysis is the most important step of coal conversion process, and the thermal processing of coal is considered as the main source of PAHs in the environments [2]. The generation and emission control of PAHs in the coal transforming processing were focused on by many researchers. In recent years, many researchers have investigated the influence of metal oxide catalysts on PAHs yields during coal combustion and organic matter decomposition [3–7]. A prominent catalytic effect on cracking biomass oil by using olivine or dolomite was found [8–11]. Using the characteristic online coal pyrolysis and products catalytic cracking analysis technology, this paper probed into the cracking effects of CaO or Fe_2_O_3_ which were the main effective compositions of olivine or dolomite on the 16 PAHs of the coal pyrolysis products on the Environmental Protection Agency (EPA) Priority Pollutant List. The applicable catalysts and catalytic conditions reducing the PAHs emission during coal pyrolysis were selected. The results also provided a theoretical foundation for the industrial application.

## 2. Materials and Methods

### 2.1. Materials

#### 2.1.1. Coal Sample

PS coal (from Pingshuo coal mine, China) that can generate more PAHs during coal pyrolysis was chosen as the sample in the study. The particle size was between 100 and 120 mesh. The proximate and ultimate analysis of PS coal was shown in Table 1.

#### 2.1.2. Catalysts

Analytical pure calcium oxide (CaO) and andiron oxide (Fe_2_O_3_) were used as the catalysts and were crushed to 60–80 mesh, 100–120 mesh, 140–160, and 180–200 mesh, respectively.

#### 2.1.3. Chemicals and Reagents for the Experiment

The mixed standard sample of the 16 PAHs was supplied by Accustandard Inc. (concentration: 200 mg/mL; solvent, a mixture of methanol, and dichloromethane, v : v = 1 : 1).

### 2.2. Experiment Procedure

Pyroprobe CDS 5250 pyrolyser coupled with FOCUS gas chromatograph and DSQII mass spectrometer (Thermo Fisher, USA) was used in this study. The connection and detective condition of GC-MS was presented in Dong et al. [12]. In each experiment, the catalyst was placed in a quartz filler tube which was firstly filled with a long quartz filler rod designed for use with the autosampler. Then, the catalyst was placed above the coal and they were separate by some quartz wool. It is functioned as a fixed bed, so that all the pyrolysis vapors will pass through the catalyst layer. The pyrolysis temperature was set differently, with a heating rate of 10°C/ms. The coal pyrolysis products were cracked by the catalyst on catalyst layer and the products were analyzed by the GC-MS for qualitative or quantitative analysis. Helium was used as the carrier with a flow rate of 1 mL/min.

### 2.3. Quantitative Analysis of PAHs

For the convenience of investigating the catalytic effects of metallic oxidant on PAHs with different ring number, the 16 PAHs were divided into LMW-PAHs (2-3 rings PAHs): naphthalene (2 rings), acenaphthylene (3 rings), acenaphthene (3 rings), fluorene (3 rings), phenanthrene (3 rings), anthracene (3 rings), MMW-PAHs (4 rings PAHs): fluoranthene (4 rings), pyrene (4 rings), benzo[a]anthracene (4 rings), chrysene (4 rings), HMW-PAHs (5-6 rings PAHs): benzo[b]auoranthene (5 rings), benzo[k]fluoranthene (5 rings), benzo[a]pyrene (5 rings), indeo[123-c,d]pyrene (6 rings), dibenz[a,h]anthracene (6 rings), and benzo[g,h,i]perylene (6 rings) [13].

The cracking rate of 16 PAHs was defined as

(1) $$\begin{array}{c} X=\frac{C_{1}-C_{2}}{C_{1}}\times 100\%, \end{array}$$

where *C*_1_ is the concentration of 16 PAHs in pyrolysis gas and *C*_2_ is the concentration of 16 PAHs after catalytic cracking.

The quantitative analysis of 16 PAHs was performed by the external standard method. The standard curve of 16 PAHs was generated by the GC-MS using five PAHs standard solutions with different PAHs concentrations (0.2 *μ*g/mL, 0.4 *μ*g/mL, 0.8 *μ*g/mL, 1.0 *μ*g/mL, and 1.2 *μ*g/mL). The correlation coefficient (*r* ^2^) of each standard curve was greater than or equal to 0.991. In order to research the effect of metallic oxide on PAHs, the quantitative analysis of 16 PAHs was performed by mass spectrometer in SIM mode which had high selectivity and sensibility for the PAHs. Meanwhile, the qualitative analysis of pyrolysis production was performed by mass spectrometer in full scan mode to study the change of some important compounds in the pyrolysis process. All the experiments were replicated at least three times to make sure that the results were reproducible. Quality control of PAHs analysis was the same as the previous study [12].

## 3. Results and Discussion

### 3.1. The Effects of Temperature on Catalytic Cracking of PAHs

**Figure 1:**
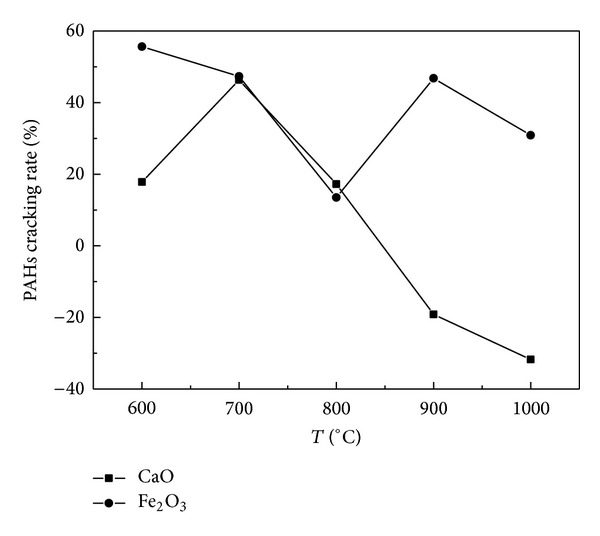
Relationship between the total PAHs cracking rate and temperature under effect of catalysts.

The influences of temperature were discussed on the emission of 16 PAHs cracked by CaO and Fe_2_O_3_ during coal pyrolysis, and the amount and the particle size of CaO and Fe_2_O_3_ were 0.4 mg and 140–160 mesh, respectively.  Figure 1 showed that CaO and Fe_2_O_3_ can reduce the total PAHs (represented the total amount of 16 PAHs). The maximum cracking rate for Fe_2_O_3_ is 55.64% at 600°C and it is better than that of CaO which is 44.29% at 700°C.

It can be inferred that there were many active centers on the CaO surface. These active centers could deform *π*-electron cloud of the condensed aromatic compounds generated from the coal pyrolysis [14]. That is to say, the stability of PAHs was weakened and the activation energy of catalytic cracking PAHs was lowered. It illustrated that CaO was advantageous in cracking PAHs.

**Figure 2:**
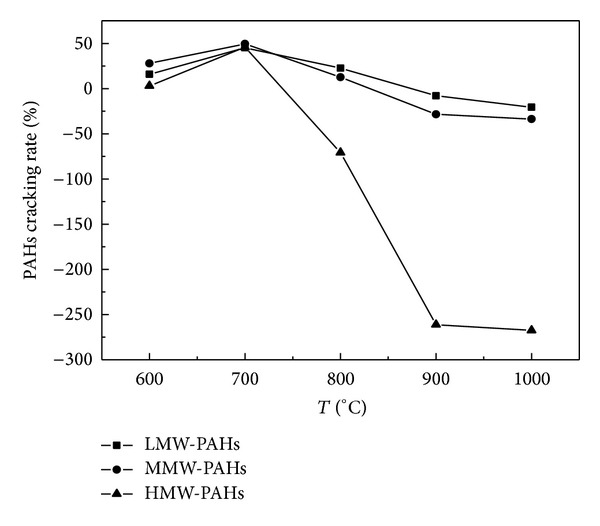
Relationship between the rings cracking rate distribution of PAHs and temperature under effect of CaO.

When the temperature is higher than 900°C, total account of PAHs from catalytic cracking coal pyrolysis products was higher than that generated from the raw coal pyrolysis. As is shown by Figures 1 and 2, the PAHs cracking rate increased with the increase of temperature, especially that of the HMW-PAHs. At a high temperature (900–1000°C), CaO played a major role in PAHs dehydrogenization which led to a polycondensation of PAHs [15], generating more PAHs from the catalytic coal pyrolysis products.

So the conclusion was that CaO had different catalytic roles at different temperatures for PAHs. At the high temperatures, CaO would lose the capability of decreasing 16 PAHs. At the low temperatures (600–800°C), CaO played a major role in catalytic cracking of PAHs.

Compared with CaO, Fe_2_O_3_ was observed to have the cracking effect on PAHs at different temperatures (600°C, 700°C, 800°C, 900°C, and 1000°C), but the cracking rate for total PAHs did not reveal a principle tendency with the increase of the temperature. At 800°C, the total PAHs cracking rate was the minimum for Fe_2_O_3_. Some possible reasons are that the coal pyrolysis process released CO and H_2_ reacting with Fe_2_O_3_ and the products Fe_3_O_4_, FeO, and Fe or other materials were generated and had an effect on catalytic cracking of PAHs [16].

**Figure 3:**
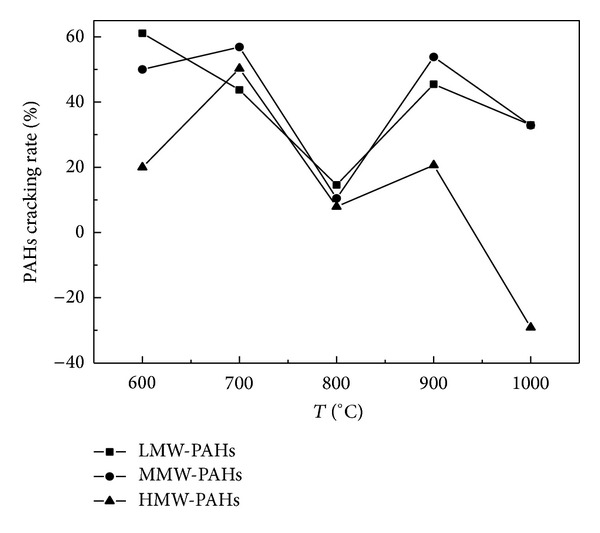
Relationship between the rings cracking rate distribution of PAHs and temperature under effect of Fe_2_O_3_.

By comparing Figures 2 and 3, it can be found that the cracking capacity of Fe_2_O_3_ on LMW-PAHs and MMW-PAHs was better than that of CaO at a temperature in the range of 600°C–700°C. CaO exposed a better cracking capacity on LMW-PAHs at 800°C than Fe_2_O_3_, and the corresponding cracking capacity on MMW-PAHs for the two catalysts was similar. When the temperature was higher than 900°C, Fe_2_O_3_ exposed cracking effect on LMW-PAHs and MMW-PAHs while CaO had the catalytic forming effect on all the PAHs. Besides, a more remarkable catalytic forming effect on HMW-PAHs was found at 1000°C by CaO.

### 3.2. Effects of Particle Size during Catalytic Cracking of PAHs

In order to reveal the influence of catalysts particle sizes on the catalytic cracking effect, different particle sizes (60–80 mesh, 100–120 mesh, 140–160 mesh, and 180–200 mesh) were selected for a fixed amount of catalyst. Temperatures of 700°C and 600°C were chosen which were the highest cracking rate for CaO and Fe_2_O_3_, respectively. The amount of catalysts was set to 0.4 mg.

**Figure 4:**
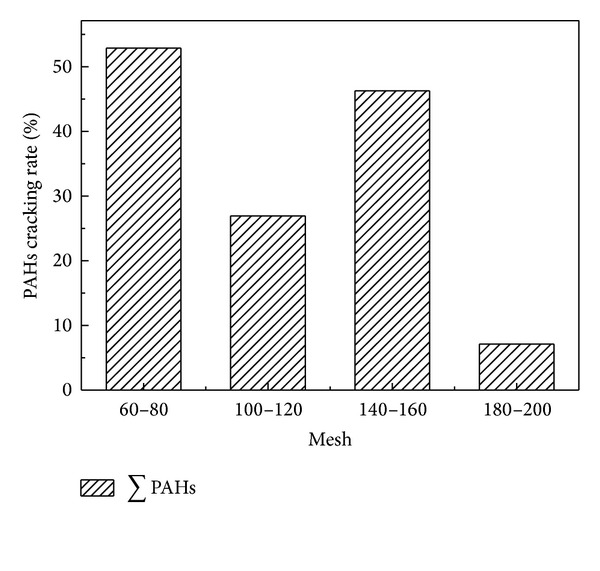
Relationship between the total PAHs cracking rate and particle size of CaO.

**Figure 5:**
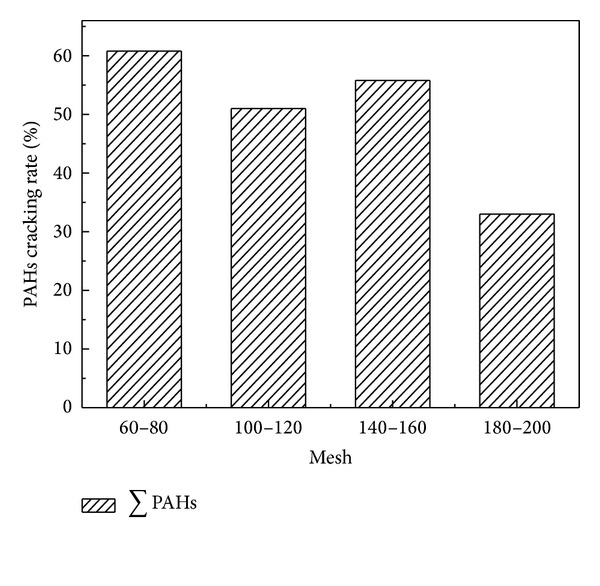
Relationship between the total PAHs cracking rate and particle size of Fe_2_O_3_.

**Figure 6:**
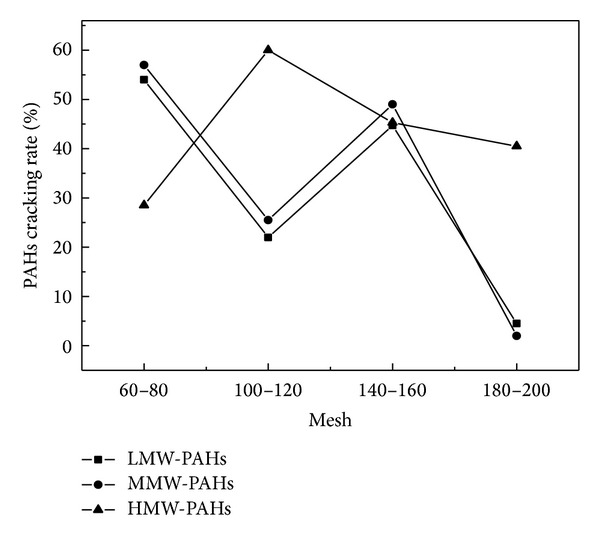
Relationship between the rings cracking rate distribution of PAHs and particle size of CaO.

**Figure 7:**
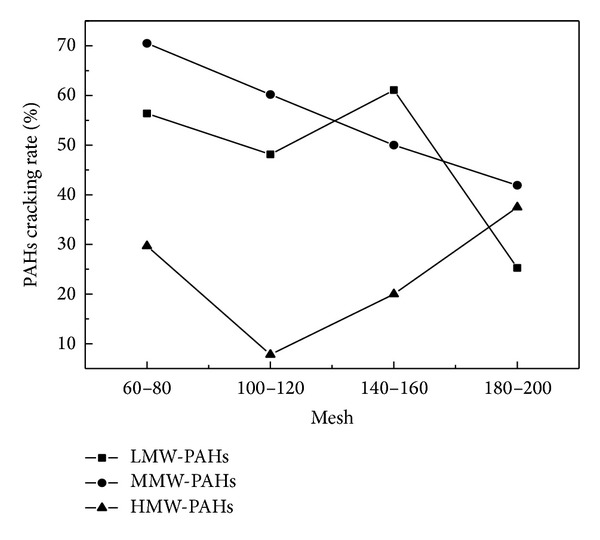
Relationship between the rings cracking rate distribution of PAHs and particle size of Fe_2_O_3_.

Figures 4 and 5 demonstrated that the total PAHs cracking rate decreased with the decrease of catalyst particle size. The maximum cracking rate of PAHs was 52.88% and 60.59%, respectively, for CaO and Fe_2_O_3_ with 60–80 mesh. Figures 6 and 7 showed that the cracking rate of LMW-PAHs and MMW-PAHs declined with the increase of particle size through CaO or Fe_2_O_3_, while the HMW-PAHs cracking rate had a contrary tendency. The reason for these phenomena could be that the cracking activity of two catalysts increased with the increase of particle size. The organic macromolecular compounds cracked into the LMW-PAHs, MMW-PAHs, or gas products through catalysts, which cause an increase of LMW-PAHs and MMW-PAHs during coal pyrolysis. As a result, the cracking rate of LMW-PAHs and MMW-PAHs was decreased.

### 3.3. Catalytic Cracking Mechanism

To deeply understand the catalytic cracking effect on coal pyrolysis products and find out possible process formed or cracked the PAHs, the distribution of catalytic cracking products was analyzed in the GC-MS on full scan mode. This experiment used CaO and Fe_2_O_3_ as catalysts with 0.4 mg and 140–160 mesh, respectively. CaO performed a catalytic cracking effect on 16 PAHs at 700°C and a forming effect at 900°C. But Fe_2_O_3_ only showed a catalytic cracking effect on 16 PAHs. So, 700°C was selected for the contrast.

**Figure 8:**
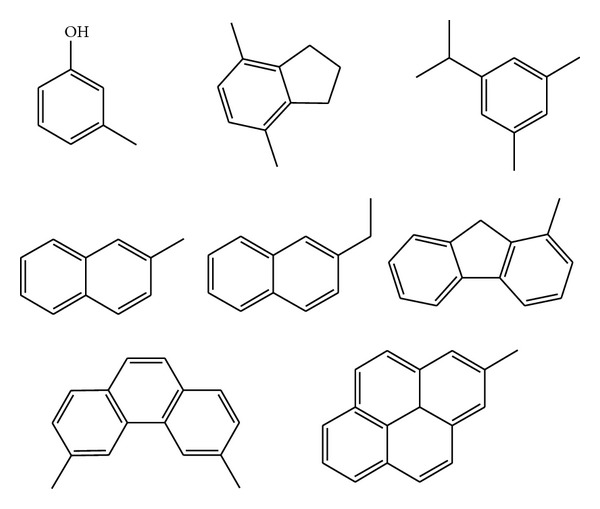
Portion products from coal pyrolysis.

The LMW-PAHs, MMW-PAHs and their isomers was shown in Figure 8, and they were catalyzed by CaO at 700°C as mentioned above. In addition, the PAHs and their derivatives were decreased synchronously. It was illustrated that CaO had an effect on breaking both the aromatic rings of PAHs, aromatic hydrocarbons with branches and aliphatic hydrocarbons. With the amount of naphthalene decreased by CaO, the alkyl-substituent benzene also decreased. It was known that naphthalene is more favorable to transforming into the gas, small molecular alkanes and alkenes, alkylene-substituent benzene, or their isomers.

**Figure 9:**
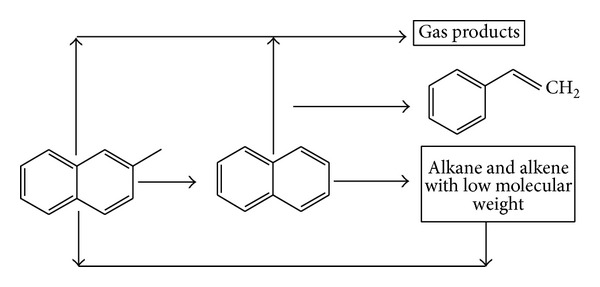
The schematic diagram of generating or cracking of naphthalene.

In the case of methylnaphthalene, Figure 9 showed the probable path to forming and cracking of PAHs with CaO. The compound on the left represents the aromatic ring with branches, the naphthalene in the middle is aromatic ring, and the ethenylbenzene is the aromatic ring with alkenyl.

**Figure 10:**
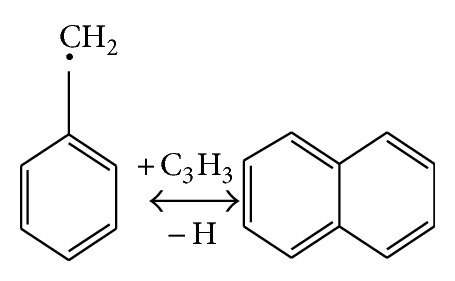
The polycondensation of methylbenzene.

At 900°C, CaO played a major role in PAHs dehydrogenation and catalyzed the coal pyrolysis products to polymerize. Taking methylbenzene as an example, when the temperature was higher, the methylbenzene should lose a hydrogen atom, then become the free radical; the free radical reacted with other lower weight molecules and formed naphthalene. On the contrary, the naphthalene and the methylbenzene increased. It was illuminated that the amount of naphthalene that came from free radical reaction was lower than that from other macromolecules cracked during the catalytic cracking process; the catalytic effect of CaO did not facilitate the naphthalene formation reaction shown in Figure 10. Also referring to Figure 2, CaO had a more significant forming effect on HMW-PAHs at 900°C, but less on medium-low-ring. It shows that CaO was possessed of selectivity on the polycondensation of PAHs.

Results of the experiment showed that the aromatic ring with aliphatic group could be transformed by Fe_2_O_3_, and some phenols such as phenol and cresol were formed after the catalytic cracking of 16 PAHs by Fe_2_O_3_. The likely reason was that many reactive oxygen species (O_2_^−^, O_2_^2−^, O^2−^) existed on the surface of metallic oxide [17]; these reactive oxygen species would crack the 16 priority controlled PAHs to some phenols.

## 4. Conclusions

Fe_2_O_3_ plays an obvious catalytic role than CaO in reducing PAHs emission during coal pyrolysis. Their catalytic temperature at which the splitting rate reaches its maximum is different, that is, 60.59% at 600°C for Fe_2_O_3_ and 52.88% at 700°C for CaO, respectively.

Fe_2_O_3_ only shows a cracking effect on 16 PAHs and has better catalytic cracking effect for medium-low-ring PAHs than that of CaO in the temperature range of 600°C to 1000°C. The cracking effect on PAHs of CaO is predominant at medium-low temperature. At higher temperature that effect is converted to promote the forming of 16 PAHs, which may be caused by more macromolecular PAHs split.

The cracking rate of 16 PAHs increases with the increase of the particle size of CaO and Fe_2_O_3_. This increase in the cracking rates mainly contributes to the medium-low-ring PAHs and the cracking rate of higher-ring PAHs tends to decrease.

The possible cracking process of PAHs from coal pyrolysis products is proposed by analyzing elaborately the cracking effect of the two catalysts on the 16 different PAHs species and comparing the composition difference of the pyrolysis products before and after the catalytic cracking. Some phenols may form after the catalytic cracking of 16 PAHs by Fe_2_O_3_.

## Figures and Tables

**Table 1:** Proximate and ultimate analysis of PS coal.

Proximate analysis wt/%			Ultimate analysis wt/%, daf				
*M*_ad_	*A*_*d*_	*V*_daf_	C	H	O*	N	S
2.2	18.3	37.2	80.4	5.2	11.9	1.4	1.1

ad: air dried basis.

*d*: dried basis.

daf: dry and ash-free basis.

*By difference.
